# Expanding the phenotypic spectrum of Xq28 duplication involving MECP2: a familial case report

**DOI:** 10.3389/fpsyt.2026.1810637

**Published:** 2026-04-22

**Authors:** Katerina Gaberova, Iliyana Hristova Pacheva, Ralitsa Yordanova, Tihomir Todorov, Albena Todorova, Liliyana Grozdanova, Margarita Panova, Mariyana Georgieva, Ivan Stefanov Ivanov

**Affiliations:** 1 Pediatrics Department, Medical University Plovdiv, Plovdiv, Bulgaria; 2 Genica Laboratory, Sofia, Bulgaria; 3 Medical Center Raredis, Plovdiv, Bulgaria; 4 Pediatrics Department, University Hospital Prof. Dr. Ivan Kirkovich, Stara Zagora, Bulgaria

**Keywords:** gene dosage effect, intellectual disability, intrafamilial variability, *MECP2* duplication syndrome, Xq28 duplication

## Abstract

X-linked intellectual disability (XLID) is a well-recognized group of neurodevelopmental disorders, with pathogenic variants in X-chromosomal genes accounting for approximately 16% of intellectual disability cases in males. Clinical expression in females is variable and depends on patterns of X-chromosome inactivation. We describe three affected individuals from a single family with XLID caused by a confirmed duplication of the Xq28 region, including the genes *SLC6A8, L1CAM, MECP2, TKTL1, FLNA*, and *GDI1*. Two male siblings presented with severe phenotypes, including profound intellectual disability, severe speech impairment, behavioral issues, facial dysmorphism, spastic cerebral palsy, epilepsy, and cutaneous abnormalities. Their mother showed mild intellectual disability and skin manifestations. Family history suggested additional affected male relatives with a similar or even more severe clinical presentation. The duplication of multiple dosage-sensitive genes within the Xq28 region likely explains the multisystem involvement and the marked phenotypic variability observed between male and female family members. This report highlights the importance of considering Xq28 duplication, the most common X-linked copy number variation associated with intellectual disability, in the differential diagnosis of families with X-linked intellectual disability, especially if it is accompanied by additional neurological impairment.

## 1 Introduction

Xq28 duplication syndrome is a rare chromosomal disorder caused by the duplication of a segment on the long (q) arm of the X chromosome, particularly involving the region at cytogenetic band Xq28 with currently about 82 individuals described in the literature (1). This duplication results in functional disomy for the duplicated genes — the affected individuals have an extra copy of the genetic material and, consequently, increased gene dosage. The condition is part of a broader group of X-linked genomic disorders with substantial neurodevelopmental and systemic effects and is increasingly recognized thanks to advances in molecular diagnostic techniques such as array comparative genomic hybridization (array-CGH) and high-resolution chromosome microarrays (2).

A critical contributor to the clinics in most cases is the duplication of the *MECP2* (methyl-CpG binding protein 2) gene, which is located within Xq28 and plays an essential role in regulating gene expression in the brain. Increased dosage of *MECP2* — typically through duplication rather than point mutation — leads to a distinctive clinical phenotype, particularly in males, manifesting as moderate to severe intellectual disability, delayed psychomotor development, hypotonia, and impaired speech, often alongside recurrent infections and autistic features (3). *MECP2* duplication is wildly discussed in the literature with hundreds of affected males reported worldwide unlike the broader spectrum of Xq28 duplication involving other genes.

The clinical manifestations of Xq28 duplication are influenced by sex and X-chromosome inactivation. In males (who have a single X chromosome), the presence of an Xq28 duplication leads directly to overexpression of the duplicated genes and a severe neurodevelopmental delay. In contrast, females typically have two X chromosomes; preferential inactivation (“skewed X-inactivation”) of the duplicated chromosome often reduces phenotypic severity. However, a subset of females with random X-inactivation or with duplications arising *de novo* or as part of more complex chromosomal rearrangements can exhibit significant cognitive and behavioral symptoms (4).

Case series and cohort studies show that the Xq28 duplication syndrome phenotype is variable in severity, with some individuals demonstrating profound impairments in motor skills and cognition, while others present with milder neuropsychiatric manifestations. Additional genes within the duplicated segment — such as *SLC6A8, L1CAM, RAB39B, TKTL1, FLNA, GDI1* — may contribute to the variability in clinical presentation, although the exact genotype-phenotype correlations remain under investigation (5). This further deepens the clinics of the patients with additional brain anomalies, muscle tone changes (hypotonia or spasticity), epilepsy.

Overall, Xq28 duplication syndrome exemplifies how alterations in gene dosage, rather than simple loss-of-function mutations, can disrupt developmental pathways and neural function. There are still uncertainties about the full phenotypic spectrum, including among individuals from the same family. Depending on the severity of the neurological deficit, some of the patients might also mimic cerebral palsy.

## 2 Aim of the report

To describe a family with a genetically confirmed duplication of the Xq28 region involving the genes *SLC6A8, L1CAM, MECP2, TKTL1, FLNA*, and *GDI1*, characterized by marked intrafamilial phenotypic variability, including dysmorphic features, cutaneous abnormalities, and intellectual and neurological impairment of varying severity.

## 3 Materials and methods

Three members of the same family were included in the study - two male siblings with severe neurological involvement and their mother with a milder clinical phenotype. Both children were admitted to the Neurology Unit of the Pediatric Department at our tertiary care center and underwent comprehensive clinical assessment using a standardized diagnostic algorithm for familial intellectual disability. Additionally, the mother underwent a clinical examination and intelligence quotient (IQ) evaluation. Detailed genealogical analysis revealed six additional affected male relatives on the maternal side of the family, which are not included in this study. MLPA (Multiplex Ligation-dependent Probe Amplification) analysis targeting the Xq28 region was performed in all three patients described in this case report. The analysis was conducted in a diagnostic laboratory (Genica, Sofia, Bulgaria) using a laboratory-validated probemix targeting genes within this region, including *SLC6A8, L1CAM, MECP2, TKTL1, FLNA*, and *GDI1*. The results confirmed the presence of an Xq28 duplication in all three patients. Written informed consent was obtained from the parents of the participants for inclusion in the research project. All procedures performed in the studies were done in accordance with the ethical standards of the institutional research committee.

## 4 Case presentation

### 4.1 Case 1

The first patient, included in this scientific investigation, is a male, born from the fourth pregnancy, which was uneventful, following a normal vaginal delivery. Birth anthropometrics were within normal limits (birth weight 3,200 g; length 52 cm). There was no evidence of perinatal asphyxia, and the neonatal period was unremarkable.

From early infancy, global delays in neuropsychological development were evident. In the gross motor domain, rolling over was achieved at approximately 7 months of age but remained difficult. Independent sitting with crossed legs was attained around 12 months. He never achieved standing or independent ambulation. Fine motor development was markedly impaired.

Neurocognitive and social development were also severely delayed. He began to visually attend to and recognize familiar people after 1 year of age. Since infancy, he has produced only inarticulate vocalizations; from approximately 9 years of age, he began using repetitive syllables (“ma-ma,” “ta-ta”). He is able to smile and wave goodbye, and displays frequent stereotyped, monotonous shaking movements. Prior to 15 years of age, he had not been evaluated by a pediatric neurologist. His condition had been labeled as “spastic cerebral palsy” in medical records by his general practitioner.

From the age of 13 years, his parents observed episodic behavioral changes characterized by brief (10–20 s) episodes of impaired awareness with staring and a “sleepy” appearance, consistent with atypical absence seizures. In addition, episodes of sudden head drops suggestive of atonic seizures were reported, as well as rare nocturnal tonic seizures characterized by sudden generalized stiffness with extension of the limbs and occasional vocalization. At 14 years of age, generalized tonic–clonic seizures emerged. Following seizure onset, the patient was referred to our department for further evaluation and treatment.

On physical examination, facial dysmorphic features were noted, including large, poorly shaped earlobes, low anterior hairline, and thick lips. Ophthalmologic examination revealed left-sided convergent strabismus. Muscle tone was diffusely increased with spasticity affecting all four limbs, accompanied by contractures of the knees, ankles, and elbows. The patient was able to sit independently but was unable to walk. Muscle hypotrophy of the calf muscles was evident. Deep tendon reflexes were symmetrical and markedly brisk. Bilateral Babinski signs were present. Chaotic, non-purposeful movements of the hands were observed.

The patient demonstrated limited social interaction: he visually scanned his surroundings, waved his hand to indicate greeting or farewell, and produced inarticulate vocalizations. Formal assessment revealed profound intellectual disability, with an estimated intelligence quotient below 20.

Electroencephalography (EEG) showed impaired background activity with multifocal and generalized epileptiform discharges, including spike–slow-wave and polyspike–slow-wave complexes.

**Figure 1:**
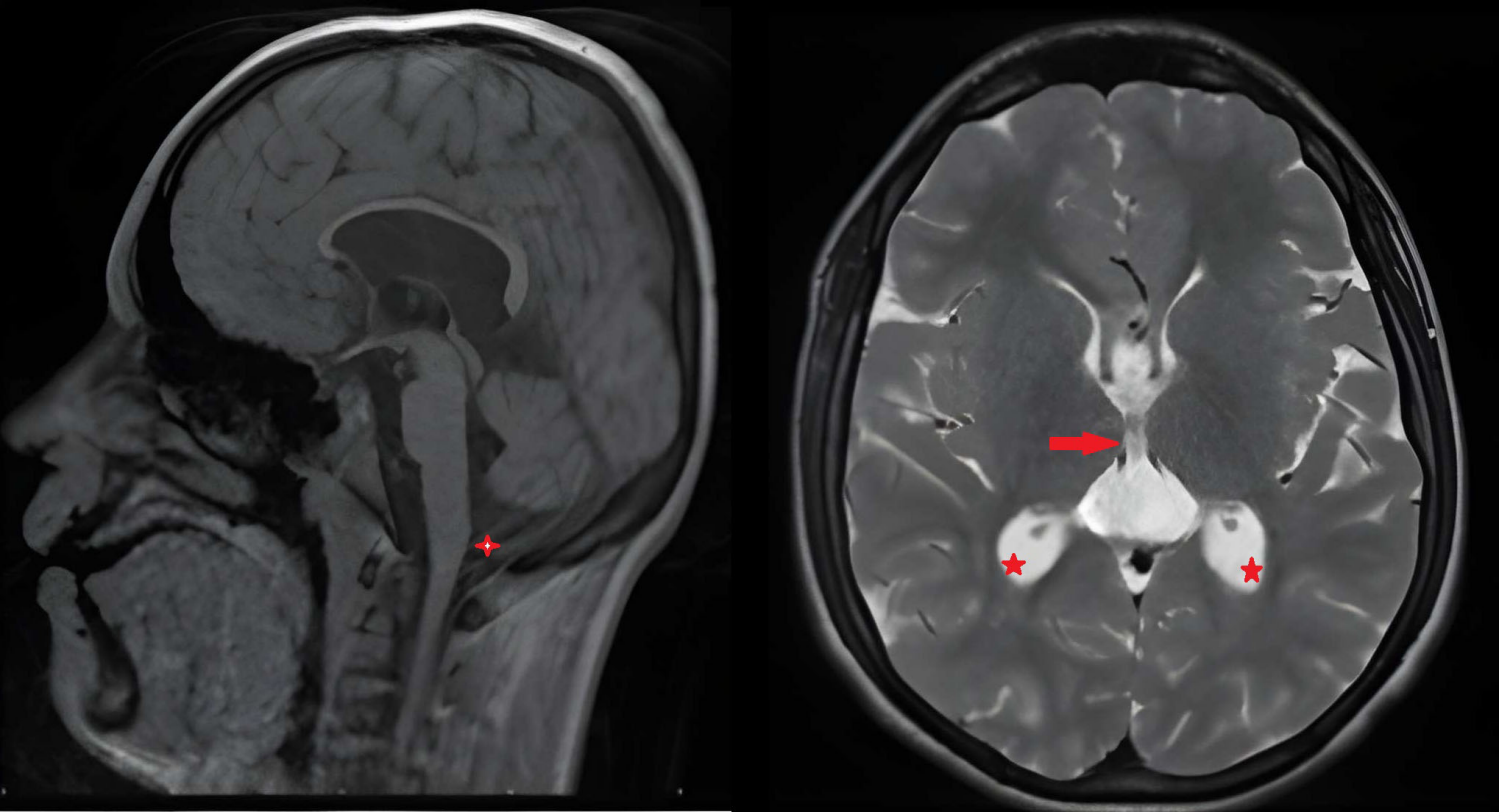
Sagittal T1-weighted and axial T2-weighted brain MRI of Case 1 demonstrating enlargement of the cisterna magna/cisterna magna permagna (indicated by a cross +), dilation of the third ventricle with communication with the cavum vergae (indicated by an arrow →), and colpocephaly (indicated by a star ☆).

Brain magnetic resonance imaging (MRI) revealed mildly dilated lateral ventricles with enlargement of the trigones and occipital horns (colpocephaly), dilation of the third ventricle with communication with the cavum vergae, and a cysterna magna permagna. The basal ganglia were bilaterally poorly structured, with absence of normal myelination in the anterior limbs of the internal and external capsules and hypomyelination of the dorsal segments. Radially oriented gyri were observed, along with hypoplasia of the fornix and hippocampi. MRI findings are presented in **Figure 1**.

### 4.2 Case 2

The second patient included in the study, is another male child in the same family, born from the fifth pregnancy, which was complicated by vaginal bleeding during the sixth lunar month. Delivery was performed by emergency cesarean section at term due to signs of fetal distress. Birth weight was 2,100 g. The neonatal course was complicated by respiratory insufficiency requiring mechanical ventilation for several days. He was discharged at 10 days of life in stable condition with a normal general physical status.

From early infancy, convergent strabismus was noted. Since birth, segmental hypopigmentation involving the left side of the body has been present.

Neuropsychological development has been significantly delayed. Independent sitting was achieved at approximately 12 months of age. The ability to stand was attained only after initiation of physiotherapy, at 3 years and 10 months of age. Vocal development was limited to syllabic sounds beginning at 4 years and 6 months; no meaningful words have developed. He is able to visually track objects named but does not point to objects with his finger.

No epileptic seizures have been observed to date.

On physical examination, hypopigmented skin lesions were observed on the left side of the chest, left half of the back, and left upper limb, with a distribution suggestive of a dermatomal pattern. An area of hypotrichosis was noted on the frontal scalp. Cranial nerve examination revealed convergent strabismus, more pronounced in the left eye. Muscle tone was diffusely increased with spasticity, predominantly affecting the lower limbs and more pronounced on the left side, accompanied by a contracture of the left knee joint. Independent gait was not possible. Deep tendon reflexes were diffusely exaggerated, with sustained ankle clonus, more evident on the left. Abdominal reflexes were preserved. No pathological reflexes of the Babinski or Rossolimo type were elicited; plantar responses were flexor bilaterally. The patient was able to maintain stable sitting but showed no effective weight-bearing or support on the lower limbs.

EEG demonstrated normal findings. Brain MRI was not performed.

### 4.3 Case 3

The mother of the two affected children, presented as Case 1 and Case 2, was also clinically evaluated. She presented with a mild intellectual disability, with an intelligence quotient of 60 as assessed by the Wechsler Intelligence Scale. She completed basic daily activities independently and did not exhibit significant behavioral disturbances. There were no reported sensory deficits, visual or auditory impairments, or systemic abnormalities. Her medical history was otherwise unremarkable.

Neurological examination revealed no focal neurological deficits. Muscle tone, strength, coordination, and gait were within normal limits. No history of epileptic seizures, paroxysmal events, or regression was reported. Cranial nerve examination was unremarkable.

Dermatological examination revealed a large hyperpigmented skin lesion involving the entire left half of the chest and extending to the left upper limb. The pigmentation showed a clear unilateral distribution with sharp midline demarcation, suggestive of a segmental or mosaic pattern. No neuroimaging or electroencephalographic studies were available at the time of evaluation.

The clinical characteristics of the three reported cases in comparison with the phenotypic features most reported in the literature are presented in **Table 1**.

**Table 1:** Comparison of the clinical characteristics observed in the three reported cases with phenotypic features most frequently described in the literature.

Symptoms reported in Xq28 duplication	Case 1 (male)	Case 2 (male)	Case 3 (female/mother)
Neurodevelopmental delay/intellectual disability	++	++	+
Hypotonia, often progressing to spasticity	+	+	–
Pyramidal signs	++	+	–
Speech and language impairment	++	++	–
Autistic features and behavioral abnormalities	+	+	–
Epilepsy	+	–	–
Structural brain anomalies	+	NA	NA
Growth abnormalities (prenatal or postnatal)	–	+	–
Congenital anomalies	–	–	–
Facial dysmorphism	+	+	–
Skin hypopigmentation (not reported so far)	–	+	+

### 4.4 Genetic testing

**Figure 2:**
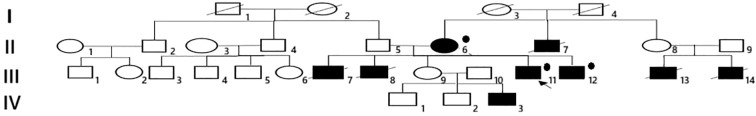
Genealogical analysis in the affected family. Roman numerals indicate generations and Arabic numbers indicate individuals. Squares represent males and circles represent females. Affected individuals are denoted by solid symbols and unaffected individuals are denoted by open symbols. The initial proband (Case 1) is indicated by an arrow, and the other participants in the DNA analysis (Case 2 and Case 3) are marked with an asterisk. Horizontal lines indicate mating, vertical lines indicate descent, and a diagonal slash denotes deceased individuals. Legend indicates: ° - Unaffected female, □ - Unaffected male, ▪ - Affected male, • - Affected female, ↖ - Proband, □──○ - Mating/partnership, ┬ - Offspring line.

Considering the clinical findings observed in the two affected siblings and their mother, a detailed genealogical analysis was undertaken. Pedigree evaluation revealed a pattern consistent with X-linked inheritance, with multiple affected male individuals across several generations of the maternal lineage. Pedigree analysis of the family is presented in **Figure 2**.

In total, eight male family members were reported to be affected by a severe neurodevelopmental disorder. These included the two boys, reported in detail (III-11 and III-12, **Figure 2**), two older male siblings born to the same parents as the index cases, both previously diagnosed with “cerebral palsy”; they died at 17 years and 4 years of age, respectively (III-7 and III-8, **Figure 2**). Additionally, two male first cousins on the maternal side, also labeled as having “cerebral palsy,” died during infancy (III-13 and III-14, **Figure 2**). A maternal uncle with a similar diagnosis died at 15 years of age (II-7, **Figure 2**). Furthermore, a male newborn born to an otherwise unaffected female family member died in infancy with a history of “severe neurological impairment” (IV-3, **Figure 2**).

Notably, no affected individuals were identified in the paternal lineage. The clustering of affected males, early lethality, and apparent absence of significant neurological manifestations in female family members strongly support an X-linked mode of inheritance, consistent with the phenotypic spectrum observed in the described cases.

The combination of a severe, early-onset neurodevelopmental phenotype in multiple male relatives, apparent female sparing or milder involvement, and a pedigree consistent with X-linked inheritance prompted further molecular genetic investigation. Given the clinical overlap among affected family members and the historical diagnoses of cerebral palsy without a clear perinatal etiology, a monogenic or contiguous gene disorder involving the X chromosome was suspected.

Subsequent genetic analysis via Multiplex-Ligation-dependent Probe Amplification (MLPA) identified a duplication of the long arm of the X chromosome at Xq28, encompassing several dosage-sensitive genes, including *SLC6A8, L1CAM, MECP2, TKTL1, FLNA*, and *GDI1*. This finding provides a unifying molecular explanation for the shared neurodevelopmental features observed.

## 5 Discussion

The presented cases from the same family describe a multigenerational X-linked neurodevelopmental disorder characterized by severe intellectual disability, spastic motor impairment, epilepsy in some affected individuals, and early male mortality. Although multiple family members were historically labeled as having cerebral palsy, the absence of a clear perinatal insult, the progressive and syndromic nature of the presentation, and the clustering of affected males along the maternal lineage all suggested an underlying genetic etiology consistent with an X-linked disorder. Subsequent MLPA analysis identified a duplication of the Xq28 region encompassing several dosage-sensitive genes, including *SLC6A8, L1CAM, MECP2, TKTL1, FLNA*, and *GDI1*, thereby providing a unifying molecular basis for the observed phenotype.

Duplications involving *MECP2* are a well-characterized cause of an X-linked neurodevelopmental syndrome, often referred to as *MECP2* duplication syndrome or X-linked intellectual disability Lubs type (OMIM: 300260). The gene encodes methyl-CpG–binding protein 2 (MeCP2), a key regulator of neuronal transcriptional activity that plays a crucial role in normal brain maturation (6). Duplication of the gene is associated with severe developmental delay/intellectual disability, absent or minimal speech, progressive spasticity, and epilepsy, particularly in affected males, consistent with previously reported clinical cohorts (7–9). The core features of *MECP2* duplication include infantile hypotonia, global developmental delay, intellectual disability, impaired or absent ambulation and speech, and recurrent infections, with epilepsy being a common comorbidity in many affected individuals (10). The two male patients involved in our study presented with a severe neurodevelopmental phenotype and clinical signs indicative of pyramidal tract dysfunction, supporting the presence of an associated structural brain anomaly. Such manifestations may be attributable to the combined dosage effects of multiple genes involved in the Xq28 duplication.

While *MECP2* is widely recognized as the principal dosage-sensitive gene within Xq28 duplications, the phenotypic variability observed among patients likely reflects the combined effects of additional duplicated genes within the region (5, 11, 12). For example, duplications that also include *L1CAM* (gene, that is responsible for neuronal migration, axonal arborization, synaptogenesis and myelination) have been associated with more severe neurodevelopmental abnormalities, including cortical dysgenesis, aqeductal stenosis and spasticity (OMIM: 308840). Several imaging findings overlap with patients with 28q duplication, including those, observed in our Case 1 (colpocephaly, cavum verge, poor myelination) (13, 14). Considering this finding, we believe that our report supports the association of the duplication with clinical manifestations, as well as with structural brain anomalies observed in some patients. Duplications of *FLNA* have been implicated in gastrointestinal and genitourinary dysfunction in some families with Xq28 duplications, despite that there is no clear evidence of triplosensitivity for the gene (https://clinicalgenome.org/, accessed on 20.03.2026). Genes such as *GDI1* and *SLC6A8* have also been independently associated with X-linked intellectual disability when mutated or altered in copy number, despite the absence of strong evidence on their triplosensitivity effect. *GDI1* plays role in neurotransmission and has been described as critical for intellectual development (15). *SLC6A8* is linked to cerebral creatine deficiency and therefore intellectual disability, behavioral disorders and mild facial dysmorphism (OMIM: 300036). Unfortunately, MRI in our index patient was performed without MR-spectroscopy, which might indicate lack of cerebral creatine peak, diagnostic for cerebral creatine deficiency. In ClinGen, there is no evidence of triplosensitivity for any of the genes individually, including *MECP2*, likely because there are no reports of patients with focal duplications of these genes (https://clinicalgenome.org/, accessed on 20.03.2026). In contrast, several regions within Xq28 show strong evidence of triplosensitivity, including *MECP2* (https://search.clinicalgenome.org/kb/gene-dosage/region/ISCA-46304) and *GDI1* (https://search.clinicalgenome.org/kb/gene-dosage/region/ISCA-37439). Multiple studies have highlighted the cumulative effect of genes within the Xq28 band on the phenotype (5, 11, 12). **Table 2** summarizes the probable impact of the genes involved in the reported duplication on male patients.

**Table 2:** Summary of the possible contribution of the affected genes on the clinics of the reported patients. .

Gene	Duplication effect
SLC6A8	May contribute to intellectual disability and epilepsy
L1CAM	May lead to structural brain anomalies
TKTL1	No certain effects
FLNA	No certain effects
GDI1	May contribute to intellectual disability and epilepsy (The region has a TS score of 3)
MECP2	Main reason for severe to profound intellectual disability, abnormalities in the muscle tone, autistic behavior (The region has a TS score of 3)

The epilepsy observed in the index patient, including atypical absence and atonic seizures with later generalized tonic–clonic seizures, is consistent with the seizure spectrum described in Xq28 duplication syndromes. The EEG findings of multifocal and generalized epileptiform discharges align with prior reports demonstrating that epilepsy in *MECP2* duplication cases can present with variable seizure types and generalized discharges (16, 17). It is considered that involvement of *GDI1* gene may play additional role for the phenotype due to its role in neurotransmission (15), although more than 50% of the reported patients with *MECP2* gene duplication also experience epilepsy (18).

Prenatal growth restriction is not classically regarded as a defining feature of *MECP2* duplication syndrome; however, it has been reported in a limited number of cases, particularly in prenatal series and in individuals with larger Xq28 duplications encompassing *MECP2* and adjacent dosage-sensitive genes (1, 19). In this context, the presence of prenatal growth restriction in our patient expands the recognized prenatal phenotype associated with *MECP2* duplication. This finding supports the view that increased dosage of genes within the duplicated Xq region may influence early growth and development, potentially contributing to *in-utero* manifestations in a subset of affected individuals. Recognition of prenatal growth abnormalities in association with *MECP2* duplication may have implications for prenatal assessment and counseling, particularly when growth restriction is identified alongside other suggestive features or a relevant family history.

The phenotype observed in the mother — consisting of mild intellectual disability with unilateral cutaneous hyperpigmentation and no major neurological deficits — underscores the influence of X-chromosome inactivation on phenotypic expression in females carrying Xq28 duplications. In many reported cases, carrier females are asymptomatic or with mild phenotype due to skewed inactivation of the duplicated X chromosome, although symptomatic females with interstitial Xq28 duplications have also been described, particularly in the context of random X-inactivation (4, 20). The unilateral hyperpigmentation in this case may represent manifesting mosaicism, reflecting tissue-specific patterns of X-inactivation that modified gene expression in affected somatic lineages (21). None of the genes, affected by the duplication, are reported to cause cutaneous manifestations.

This study contributes to the existing literature by describing three individuals with X-linked intellectual disability, including two affected male siblings and their heterozygous mother, who exhibited a milder clinical phenotype. In the male patients, prominent clinical features included global developmental delay accompanied by spasticity and pyramidal signs, which initially led to a misdiagnosis of cerebral palsy by the primary care physician. The combined dosage effects of the Xq28 genes, involved in the reported duplication, likely contribute cumulatively to the observed clinical phenotype. Several limitations should be acknowledged, including the small number of patients, limited clinical information available for other affected family members, and the inability to obtain neuroimaging studies for the second male patient and the mother. Another limitation is that the genetic analysis available through this investigation does not provide the exact size of the duplication and, consequently, cannot determine whether additional genes are affected.

## 6 Conclusion

Historically, individuals with severe neurodevelopmental impairment in infancy or childhood have often been misclassified with diagnoses such as cerebral palsy in the absence of genetic testing; such misdiagnoses likely delayed appropriate molecular evaluation in multiple affected relatives in this family. This underscores the importance of considering genomic testing, including array comparative genomic hybridization or other copy number variant (CNV) assays, in males with unexplained global developmental delay, especially when there is a suggestive family history.

In conclusion, this family illustrates the broad phenotypic spectrum associated with Xq28 duplications, highlights that *MECP2* is a critical but not exclusive contributor to clinical severity, and emphasizes the utility of genomic characterization for accurate diagnosis, prognostication, and genetic counseling. Recognition of Xq28 duplication syndromes has direct implications for recurrence risk assessment and carrier detection in at-risk female relatives.

## Data Availability

The data supporting the findings of this case report are not publicly available due to privacy and ethical restrictions but are available from the corresponding author upon reasonable request. Requests to access these datasets should be directed to katerina.gaberova@phd.mu-plovdiv.bg.
